# The interface between inhibition of descending noradrenergic pain control pathways and negative affects in post-traumatic pain patients

**DOI:** 10.3109/03009734.2011.653606

**Published:** 2012-08

**Authors:** Yulong Cui, Junmei Xu, Ruping Dai, Liang He

**Affiliations:** Department of Anesthesiology, The Second Xiangya Hospital, Central South University; Anesthesiology Research Institute, Central South University, Changsha, Hunan, People's Republic of China

**Keywords:** Descending control, noradrenaline, pain, serotonin, trauma

## Abstract

**Background:**

Animal studies have shown that surgical trauma activates the descending noradrenergic pathway. However, perioperative patients have decreased concentrations of noradrenaline (NA) in the cerebrospinal fluid (CSF). We proposed that the descending monoaminergic pathway is altered in post-traumatic pain patients and that CSF monoamine neurotransmitters may be more closely related to affective symptoms. We investigated the levels of monoamine neurotransmitters and assessed pain in these patients.

**Methods:**

Patients were divided into a post-traumatic pain group, a pain-free group, a painful labor group, and a pain-free labor group. CSF was collected from all patients, and concentrations of NA, 3-methoxy-4-hydroxyphenylglycol (MHPG), dopamine, homovanillic acid, and 5-hydroxyindoleacetic acid (5-HIAA) were measured by high-performance liquid chromatography.

**Results:**

In the post-traumatic pain group, lumbar CSF concentrations of NA and MHPG were significantly decreased (*P* < 0.01) compared to the control group. The post-traumatic pain group displayed a significant negative correlation between NA and the respective total value of the short form of the McGill pain questionnaire (SF-MPQ), SF-MPQ (affective), and visual analog scale (*r* = –0.388, *r* = –0.433, and *r* = –0.367; *P* < 0.05).

**Conclusions:**

Post-traumatic pain patients demonstrated decreased concentrations of NAin CSF, indicating that descending noradrenergic pain control pathways may be inhibited. NA is more closely related to negative affects in post-traumatic pain patients.

## Introduction

Classical neurotransmitters such as noradrenaline (NA), serotonin, dopamine (DA), and their metabolites are known as monoamines. Anatomical and physiological studies have shown that noradrenergic and serotonergic systems comprise one of the major components of descending monoaminergic pain control pathways (1). In addition to this finding, noradrenergic (2) and serotonergic (3) projections have been shown to inhibit nociceptive afferents at the level of the spinal dorsal horn neurons (4). There is growing recognition that monoaminergic neurons play a complex and crucial role as an underlying neurobiological mechanism to modulate acute and chronic pain (5).

Biogenic amines such as NA, serotonin, and DA modulate not only emotional and chronobiological processes but also the pathogenesis of affective disorders (6). It is not uncommon to find that pain perception is accompanied by affective, personality, and anxiety disorders, especially when the noradrenergic and serotonergic systems are not functioning properly (7). In addition, monoaminergic neurons control affective-emotional processes through activation of limbic structures, including the hippocampus and nucleus amygdala (8). Furthermore, these structures have interfaces with autonomic (9), visceral (10), and endocrine responses (11), which are also important in the context of depressive disorders (12). Monoamine neurotransmitters may play a critical role in connecting pain and emotion.

A few previous studies have shown that surgically induced trauma and pain can increase cerebrospinal fluid (CSF) concentrations of NA in rats and indicated that the descending noradrenergic pathway was activated (13). However, one study demonstrated that perioperative patients have a decreased concentration of NA in CSF (14). Patients with chronic and episodic pain often show depressive and affective symptoms. Moreover, decreased NA and 5-hydroxyindoleacetic acid (5-HIAA) concentrations illustrate that monoaminergic transmitters may be the interface between pain and depression (15). Because pain usually encompasses emotional aspects, in post-traumatic pain patients and in women experiencing labor pain, we hypothesize that the descending monoaminergic pathway is altered and that CSF concentrations of monoamine neurotransmitters may be more closely related to affective symptoms.

However, levels of NA, 3-methoxy-4-hydroxyphenylglycol (MHPG), DA, homovanillic acid (HVA), 5-HIAA, and 5-hydroxytryptamine (5-HT) in the CSF and affective symptoms have not been evaluated in patients with various types of acute pain. The primary aim of this study was to determine the concentrations of these neurochemical variables in order to assess the status of the descending monoaminergic pain control pathway in patients experiencing different kinds of acute pain. In addition, we evaluated the link between affective symptoms and descending pain control pathways with the short form of the McGill pain questionnaire (SF-MPQ).

## Materials and methods

### Patient selection

The patients were recruited at the Second Xiangya Hospital of Central South University. With the approval of the local Ethics Committee and written informed consent, 124 patients were enrolled. Study subjects were selected from a patient population that had received spinal anesthesia. Inclusion criteria included an age between 18 and 45 years, and an American Society of Anesthesiologists physical status of I–II, undergoing surgery with spinal anesthesia or combined spinal and epidural anesthesia. Patients with pain due to acute problems, such as trauma of the lower extremities, or an emergency cesarean section associated with labor pain, were recruited. All patients experienced pain for less than 24 h. The corresponding control groups were pain-free patients and elective cesarean section patients. Four groups of patients were studied. The first group consisted of 30 patients with sharps injuries in their lower extremities. The second group, which was the control group for the traumatic group, consisted of 33 pain-free patients undergoing elective surgery. To create a control group for the acute trauma pain group, we recruited patients with surgical diseases who were pain-free. The third group consisted of 30 at-term women undergoing painful, active labor. The fourth group consisted of 31 pain-free women having an elective cesarean section as the pain-free control group. Exclusion criteria included the use of an analgesic or any other medication preoperatively. Also excluded were patients with neuropathic pain, neurological disease, alcohol or drug abuse, or using any substances acting on the central nervous system. All patients in the four groups were premedicated with 100 mg of phenobarbital sodium intramuscularly, 30 min prior to transportation to the operating room. All patients received 500 mL of Ringer's lactate solution before anesthesia induction.

### Pain evaluation

Before surgery, patients completed a questionnaire using the visual analogue scale (VAS). Each patient used a 10-cm visual analog scale and the SF-MPQ in the shortest possible time. VAS scores and SF-MPQ were recorded preoperatively before lumbar puncture for spinal anesthesia.

### CSF sampling and neurotransmitter measurement

Samples were collected with subjects placed in the lateral position. The epidural space was identified at the L2–L3 or the L3–L4 interspace. Twenty minutes before lumbar puncture, a eutectic mixture of local anesthetic cream was applied to the lumbar area of the back to anesthetize the puncture site. After intradermal injection of 1% lidocaine, a 20-gauge introducer needle was used to penetrate the skin and superficial tissue. Epidural attempts were performed by anesthesiologists using an 18-gauge or 16-gauge Tuohy epidural needle. The loss-of-resistance-to-saline technique was used. The introducer was used because the Sprotte 90-mm 25-gauge pencil point spinal needle is very thin and has a relatively dull tip. The spinal needle was then inserted with the introducer through the L2–L3 or L3–L4 interspace and into the spinal canal. After obtaining free flow of the CSF, a total of 2 mL of CSF was collected. The fluid was immediately divided into 1-mL aliquots and frozen at –70°C until monoamines and their end-products were assayed.

The contents of the CSF were determined as previously described (16). After treatment with 0.1 M perchloric acid, the CSF samples were centrifuged at 4°C (15,000 × *g*) for 10 min. Acid supernatants were collected and measured by a high-performance liquid chromatography separation column and a 4-sensor coulometric electrochemical detector (ESA Co., Bedford, MA). Levels of NA, MHPG, DA, HVA, and 5-HIAA in the CSF were determined.

### Statistics

Data are presented as a mean ± standard deviation (SD) or standard error of mean (mean ± SEM). Even after logarithmic transformation, CSF neurochemical variables were not normally distributed for the VAS and SF-MPQ, as well as for neurotransmitter concentrations. Therefore, these values are presented as median ± 25th and 75th percentiles. Non-parametric tests were used for the analyses. The influence of variables, such as height, weight, age, and sex, on the concentration of neurotransmitters was determined by a Mann–Whitney rank sum test. When comparing more than two group means, the non-parametric Kruskal–Wallis test was used. Because there was an α-error and a potential for inflation, a *post-hoc* test using the Bonferroni correction was calculated for paired comparison. To calculate correlations between different variables, Spearman's correlation coefficient was used. A *P* value of < 0.05 was considered statistically significant. Statistical analyses were performed using the statistical program PASW statistics 18^©^.

## Results

### Patient characteristics

The demographic variables, given in Table I, did not differ among groups, with the exception of decreased height (*P* < 0.05) in pregnant women compared to other groups. Although women in active, painful labor and women receiving an elective cesarean section were shorter than the control group, which consisted of an equal mixture of men and women, they were not shorter than the female trauma and pain-free patients (Table I). The post-traumatic pain patients (18 males, 12 females) had a mean age of 31.5 years (range 20–45 years), a mean pain duration of 9.8 h (range 3.0–24 h), and reported VAS pain scores of 6.75 (3.6,7.6) (all VAS pain scores and SF-MPQ pain scores were represented as median (percentiles 25, 75)). The 33 pain-free patients (19 males, 14 females) had a mean age of 32.9 years (range 21–43 years). Thirty female patients with active, painful labor had a mean age of 29.8 years (range 24–37 years), a mean duration of labor pain of 9.6 h (range 2.9–21), and reported VAS pain scores of 6.2 (4.8,7.6). The 31 pain-free patients serving as the control group to the labor pain group had a mean age of 30.3 years (range 24–40 years). These patients were undergoing elective cesarean section and were not experiencing any labor pain.

**Table I.** Demographic characteristics of study subjects (mean ± SEM).

Group	*n*	Age (y)	Height (cm)	Weight (kg)	Sex (M/F)	Pain intensity (VAS)^a^	Pain duration (h)
Trauma	30	31.5 ± 1.2	165.5 ± 1.4	63.2 ± 1.9	18/12	6.75 (3.6,7.6)	9.8 ± 1.3
Pain-free group	33	32.9 ± 1.1	165.6 ± 1.4	64.1 ± 1.7	19/14	0	0
Painful active labor	30	29.8 ± 0.6	159.1 ± 0.4^b^	68.8 ± 0.8^c^	0/15	6.2 (4.8,7.6)	9.6 ± 1.1
Elective cesarean section	31	30.3 ± 0.8	159.5 ± 0.7^b^	69.2 ± 0.8^c^	0/15	0	0

^a^Median (percentiles 25, 75).

^b^ *P* < 0.05 versus trauma group and pain-free group, but not different from female patients.

^c^ *P* < 0.05 versus trauma group and pain-free group, using a separate analysis compared to the female patients.

### CSF monoaminergic transmitters and metabolite concentrations

The methods used in this study were reliable. The minimum measurement limits were 1.67 ng/mL (NA), 1.05 ng/mL (MHPG), 0.59 ng/mL (DA), 0.70 ng/mL (5-HIAA), and 0.85 ng/mL (HVA). The average recovery ranged from 89.5% to 104.6%. All the investigated calibration curves were characterized by a high correlation coefficient; the correlation coefficient of the five substances was 0.9943 (NA), 0.9965 (MHPG), 0.9973 (DA), 0.9991 (5-HIAA), and 0.9932 (HVA). The intra-assay precision (RSD, relative standard deviation) of NA, MHPG, DA, HVA, and 5-HIAA was 0.9%–1.2%, 1.1%–1.3%, 0.7%–1.0%, 0.8%–1.1, and 1.0%–1.3%, respectively. The inter-assay precision was 1.6%–2.7%, 0.8%–1.7%, 0.9%–1.4%, 1.2%–2.4, and 1.8%–2.8%, respectively.

**Figure 1.**
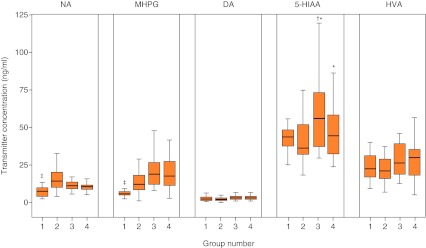
Release of monoaminergic transmitters and their end-products in CSF. Group 1 is post-traumatic pain group, group 2 is pain-free group, group 3 is painful active labor group, and group 4 is elective cesarean section group. NA= noradrenaline; MHPG = 3-methoxy-4-hydroxyphenylglycol; DOP = dopamine; 5-HIAA = 5-hydroxyindoleacetic acid; HVA = homovanillic acid. ^‡^ *P* < 0.01 versus group 2, 3, 4; *P < 0.05 versus group 2; ^†^ *P* < 0.01 versus group 4.

As a primary outcome measure, median CSF levels of monoaminergic transmitters and metabolite concentrations in the four groups (post-traumatic pain, without pain, with labor pain, and without labor pain) are presented in Figure 1. Quartiles and medians describe the general shape of the distribution of these neurochemical variables, particularly their asymmetry.

In patients with post-traumatic pain, compared to those in the pain-free control group, lumbar CSF concentrations of NA and MHPG were significantly decreased (*P* < 0.01). In the active, painful labor or the elective cesarean section group, 5-HIAA was significantly higher than in the pain-free group (*P* < 0.05), and NE and MHPG showed no significant change. Furthermore, there was a significant difference between the mean levels of 5-HIAA in the active, painful labor group and the elective cesarean group (*P* < 0.01).

### Pain assessment results

The results of pain assessment are shown in Table II. Patients in the post-traumatic pain group evaluated their pain before lumber puncture as severe (VAS 6.75 (3.6,7.6)), whereas those in the pain-free patient group had no on-going pain (VAS 0). Women in the painful labor group also evaluated their pain before lumbar puncture as severe (VAS 6.2 (4.8,7.6)). However, the elective cesarean section group reported no pain (VAS 0). There was no significant difference between the two painful groups, as their pain scores were the same.

**Table II.** Pain assessment results.

Group	SF-MPQ(*t*)	SF-MPQ(*s*)	SF-MPQ(*a*)	VAS
Trauma	19.0 (8.75,24)	14.5 (6,18)	4.5 (2,7)^a^	6.75 (3.6,7.6)
Painful active labor	18 (12,22)	14 (9,17)	3 (2,4)	6.2 (4.8,7.6)

All VAS pain scores values and SF-MPQ pain scores values were represented in median (percentiles 25, 75). ^a^ *P* < 0.05 versus active, painful labor group. SF-MPQ = short form of the McGill pain questionnaire; *t* = aggregate value of the SF-MPQ; *s* = sensory items of SF-MPQ; *a* = affective items of SF-MPQ; VAS = visual analogue score.

There was no significant difference in the SF-MPQ scores between patients with post-traumatic pain (19.0 (8.75,24)) and patients with on-going labor pain (18 (12,22)). The affective score of the SF-MPQ in patients with post-traumatic pain was significantly higher than the score in the labor pain group (4.5 (2,7) versus 3 (2,4); *P* < 0.01). However, there was no significant difference in the sensory score of the SF-MPQ between the two groups (14.5 (6,18) versus 14 (9,17)).

### Correlations between pain, affective symptoms assessment, CSF monoaminergic transmitters, and metabolite concentrations

In patients with acute post-traumatic pain, a significant decrease in NA and MHPG was detected (Table III). In this pain group, a negative correlation was detected between NA and the total value of the SF-MPQ, SF-MPQ (affective), and VAS (*r* = –0.388, *r* = –0.433, and *r* = –0.367; *P* < 0.05). In the labor pain group, only 5-HIAA had a significant positive correlation with the SF-MPQ (total), SF-MPQ (sensory), SF-MPQ (affective), and VAS (*r* = 0.463, *r* = 0.439, *r* = 0.479, and *r* = 0.418; *P* < 0.05). Other neurochemical variables showed no significant correlations with the SF-MPQ (total), SF-MPQ (sensory), and VAS. Thus, our results indicate that depressive symptoms displayed a certain degree of correlation with NA and 5-HIAA in the trauma group.

**Table III.** Correlation analysis of monoaminergic transmitters and metabolites with pain assessment.

	NA	MHPG	DOP	5-HIAA	HVA
Trauma					
VAS	–0.367^a^	–0.100	0.231	0.316	–0.068
SF-MPQ(*t*)	–0.388^a^	–0.103	0.225	0.307	–0.132
SF-MPQ(*s*)	–0.357	–0.068	0.225	0.319	–0.146
SF-MPQ(*a*)	–0.433^a^	–0.147	0.202	0.299	–0.112
Painful active labor					
VAS	0.102	0.119	–0.021	0.418^b^	0.005
SF-MPQ(*t*)	0.181	0.096	–0.057	0.463^b^	0.087
SF-MPQ(*s*)	0.173	0.086	–0.062	0.439^b^	0.100
SF-MPQ(*a*)	0.226	0.016	–0.132	0.479^b^	0.141

Correlations coefficients on a comparison between monoaminergic transmitters (and metabolites) and pain assessed as score values. ^a^ *P* < 0.05.

^b^ *P* < 0.01. SF-MPQ = short form of the McGill pain questionnaire; *t* = aggregate value; *s* = sensory items; *a* = affective items; VAS = visual analogue score; NE = norepinephrine; MHPG 3-methoxy-4-hydroxyphenylglycol; DOP = dopamine; 5-HIAA = 5-hydroxyindoleacetic acid; HVA = homovanillic acid.

## Discussion

Although the lumbar puncture itself can lead to immediate changes in concentrations of various neurotransmitters or their metabolites (17), using this method to determine the concentrations of these substances has been validated *in vivo*. Evaluating the various concentrations of neurotransmitters may provide additional information that will help put together the neurochemical processes occurring at the time the sample was taken. Many CSF transmitter studies have been performed in psychiatric disorders, such as depression (18), or in different pain models, such as labor pain (19), neuralgia (20), and postoperative pain (13). Earlier studies evaluating monoaminergic functions usually selected metabolites of monoaminergic transmitters, such as MHPG, HVA, and 5-HIAA (20), as research variables. Evaluating these variables can help determine their neurological functions. Monoamine neurons have a close relationship to pain and emotion in anatomical structures. A network of descending pathways projecting from the cerebral structures to the dorsal horn plays a complex and crucial role in pain. The locus ceruleus sends projections directly to dorsal horn neurons, and it may also receive input from the periaqueductal gray matter (21). Structures such as the locus ceruleus and the nucleus raphe magnus and neurotransmitters such as NA and 5-HT are associated with the descending control of pain. They are also closely linked to emotions such as depression, anxiety, and fear and psychological stress such as schizophrenia, anxiety disorders, personality disorders, and affective disorders. Generally, a painful event would cause fear and stress, which have a significant impact on many transmitter systems. The endogenous transmitters might be activated, but they may also induce anxiety, affection, and, in later chronic stages, depression. Various psychiatric disorders and behavioral affective disorders are implicated in the disturbances of the serotonergic and noradrenergic systems (22).

We found that there was a decreased CSF NA and MHPG concentration in post-traumatic pain patients. These patients usually experienced moderate affective symptoms. However, in the labor pain group, NA showed no significant changes. The reduction of NA is more closely linked to the affective dimension of post-traumatic pain than the sensory dimension. Our research showed a relationship between affective symptoms and pain, and the correlation coefficient (*r* = 0.433) was not low. Strittmatter et al. (15) showed that NA, serotonin, and dopamine did not change significantly in patients with acute pain. This finding may be explained by the fact that different patients were selected in the acute pain group. Patients were selected with pain lasting no more than one month in the acute pain group, and the pathogenesis of the acute pain was not clearly indicated. We limited the acute pains to 24 h after the injury. Obviously, the two types of acute pain were completely different. Even if they were acute in their characteristics, acute pain, acute post-traumatic pain, and labor pain had significant differences in CSF NE and 5-HIAA concentrations. We think post-traumatic pain and postoperative pain were more similar. Thus, differing types of acute pain may have different internal mechanisms. Oehmke et al. (14) found decreasing perioperative concentrations of NA in CSF, but they did not analyze the relationship between type of pain and the CSF concentration of NA. It is not known whether perioperative pain caused the reduction of NA. Our study showed that NA. concentration in patients with affective symptoms decreased significantly, while NA levels remained unchanged in some patients with few affective symptoms. These results support the theory that there is a relationship between depression symptoms and central decreases in monoamine system activity, which probably mediate endogenous analgesia. The state of descending control may be altered by negative emotions through complex inter-neuron connections. Animal studies showed that surgical incisional pain increases CSF concentrations of NA(13). In these studies, an incisional pain model in rats was used. This model was used to examine surgery-induced sensitization and hyperalgesia rather than model the interaction between pain and emotion (23). We believe that human post-traumatic pain and labor pain are not simple pain models, such as those used in rat models. In humans showing complex emotions, post-traumatic pain patients show more negative affective symptoms than women experiencing labor pains. Perhaps emotions are important factors that affect the monoaminergic descending pain control system. It was shown that 5-HIAA increases in patients with pain due to acute appendicitis, testicular torsion, thrombosed hemorrhoids, herniated nucleus pulposus, and ischemic pain in the lower extremities (24). However, there is no significant difference between the post-traumatic pain group and the pain-free group. Perhaps this finding is due to the unique mechanism of post-traumatic pain. Spielman et al. (25) found that CSF levels of 5-HIAA in pregnant women were significantly higher than in the non-pregnant control groups and those in active, painful labor had the highest 5-HIAA concentrations. However, in the active, painful labor group, CSF concentrations of NA and MHPG were not different from those of the cesarean section group, but 5-HIAA concentrations were positively correlated with pain. Obviously, the serotonin system was activated in patients with labor pain. Many other stressors to the brain and psychiatric disorders may initiate a ‘neurotransmitter storm’, and this observation cannot therefore be specific. Pain with a known cause, for example labor pain, might not induce affection to the same degree that post-traumatic pain does. The sample size of this study was not large, and other statistical methods to evaluate the results might have discovered a more significant conclusion. In addition, a population-based method would probably have increased information about acute pain and affective dimensions.

We observed that noradrenergic and serotonin systems showed a different, converse tendency, in post-traumatic pain, which is different from labor pain because it is usually accompanied by negative affective symptoms. These patients showed decreased NA concentrations in their CSF, indicating that descending noradrenergic pain control pathways may be inhibited. NA is more closely related to affective symptoms in post-traumatic pain patients. Negative emotions may suppress descending antinociceptive systems and promote transmission of noxious stimuli. Pregnant women may have more complex emotions, positive or negative, and their noradrenergic descending control systems are not suppressed, although serotonin antinociceptive systems are activated. The descending pain control system can inhibit nociceptive stimulus transmission.
